# Pralatrexate as a bridge to allogeneic hematopoietic stem cell transplantation in a patient with advanced-stage extranodal nasal-type natural killer/T cell lymphoma refractory to first-line chemotherapy: a case report

**DOI:** 10.1186/s13256-020-02363-3

**Published:** 2020-03-17

**Authors:** Yao-Chung Liu, Ting-An Lin, Hao-Yuan Wang, Po-Shen Ko, Chia-Jen Liu, Liang-Tsai Hsiao, Sheng-Hsuan Chien, Jyh-Pyng Gau

**Affiliations:** 1 Division of Haematology, Department of Medicine, Taipei Veterans General Hospital, No. 201, Section 2, Shipai Road, Beitou District, Taipei City, 112 Taiwan; 2 Faculty of Medicine, National Yang-Ming University, No. 155, Section 2, Linong St, Beitou District, Taipei City, 112 Taiwan; 3 Division of Transfusion Medicine, Taipei Veterans General Hospital, No. 201, Section 2, Shipai Road, Beitou District, Taipei City, 112 Taiwan

**Keywords:** Natural killer/T cell lymphoma, Peripheral T cell lymphoma, Chemotherapy, Pralatrexate, Allogeneic hematopoietic stem cell transplantation

## Abstract

**Background:**

Extranodal natural killer/T cell lymphoma, nasal type, is one of the more common subtypes of mature T cell lymphoma, especially in the Far East Asian population. This aggressive histologic subtype of peripheral T cell lymphomas is frequently susceptible to exposure of Epstein–Barr virus infection. The optimal treatment is not well elucidated. For stage IV disseminated extranodal natural killer/T cell lymphoma, induction chemotherapy with consolidative autologus or allogeneic hematopoietic stem cell transplantation is recommended as the major first-line treatment. However, there is controversy over which type of chemotherapy is most appropriate and effective as a bridge to autologus or allogeneic hematopoietic stem cell transplantation in patients with newly diagnosed disseminated advanced-stage or relapsed extranodal natural killer/T cell lymphoma because of cancer chemoresistance or associated complications. Pralatrexate is the first US Food and Drug Administration-approved novel agent for the treatment of refractory/recurrent peripheral T cell lymphomas. In our case, pralatrexate was used as a successful bridge to allogeneic hematopoietic stem cell transplantation in a patient with advanced-stage disseminated extranodal natural killer/T cell lymphoma refractory to first-line chemotherapy.

**Case presentation:**

We presented a case report of a 29-year-old Asian man diagnosed as having stage IV disseminated extranodal natural killer/T cell lymphoma, nasal type, with skin and bone marrow involvement, whose disease was primary refractory to first-line dexamethasone, methotrexate, ifosfamide, L-asparaginase, and etoposide chemotherapy, but obviously responded to treatment with two cycles of single-agent pralatrexate treatment. Monitoring Epstein–Barr virus viremia revealed dramatic downregulation. In addition to complete remission of the involvement of bone marrow and nasal cavity, skin involvement also obtained partial remission. The extranodal natural killer/T cell lymphoma successfully achieved complete remission after a bridge to allogeneic hematopoietic stem cell transplantation.

**Conclusions:**

This is the first study to present pralatrexate as a successful bridge to allogeneic hematopoietic stem cell transplantation in a 29-year-old Asian male patient with advanced-stage extranodal natural killer/T cell lymphoma refractory to first-line dexamethasone, methotrexate, ifosfamide, L-asparaginase, and etoposide chemotherapy. This case provides a novel treatment opinion for extranodal natural killer/T cell lymphoma, especially for the Far East Asian population.

## Background

Natural killer/T cell lymphoma (NKTCL) is one of the more common subtypes of mature T cell lymphoma, an aggressive subtype of peripheral T cell lymphoma (PTCL), which occurs predominantly in non-nodal sites [1, 2]. Most extranodal natural killer (NK)/T cell lymphomas (ENKL), nasal type, frequently involve the skin, soft tissue, aerogastrointestinal tract, and testis [1, 2]. ENKL is associated with Epstein–Barr virus (EBV)-positive infiltration in lymphoid tissue or viremia and occurs more commonly in Asian and South American populations than in Western populations [2–4]. The quantification of circulating plasma EBV DNA can be used to monitor the treatment response of ENKL [2–4].

Not only do patients with ENKLs have a poor survival rate at 5 years, ranging from 37.9 to 45.3%, but ENKLs also show easy resistance to chemotherapy [5, 6]. Because ENKL is often resistant to anthracycline-based chemotherapy, regimens based on asparaginase and PEG-asparaginase, such as the dexamethasone, methotrexate, ifosfamide, L-asparaginase, and etoposide (SMILE) regimen, have been effective as first-line therapy for nasal type ENKL [7, 8]. The outcomes for advanced or relapsed/refractory ENKL remain poor, however, particularly once the disease becomes refractory to first-line SMILE chemotherapy. The survival of patients receiving second-line therapy averages less than 6 months [9, 10]. Pralatrexate (PDX) is a novel drug approved by the US Food and Drug Administration for the treatment of relapsed or refractory PTCL [11]. PDX was developed as a synthetic antimetabolite folate analog and had a higher potency than methotrexate in competitively inhibiting dihydrofolate reductase. The recommended dose of PDX is 30 mg/m^2^ once weekly for 6 weeks in a 7-week cycle until disease progresses or there is unacceptable toxicity for PTCL that may require dose reduction or discontinuation [12].

Allogeneic hematopoietic stem cell transplantation (AHSCT) can be used as a first-line treatment to prevent relapse of advanced ENKL or as salvage treatment after chemotherapy [13, 14]. Guidelines from the American Society for Blood and Marrow Transplantation supported the use of both autologous hematopoietic stem cell transplantation (HSCT) and AHSCT for chemosensitive relapsed disease in localized ENKL or as a front-line consolidation therapy for disseminated ENKL [15]. However, there is controversy over which type of HSCT is most appropriate in patients with advanced-stage or relapsed ENKL. For disseminated ENKL with bone marrow involvement, it is very difficult to collect enough autologous stem cells or avoid cancer stem cell harvesting. Until now, though, there remains a lack of effective first-line treatments for newly diagnosed ENKL or a bridge to AHSCT for relapsed/refractory ENKL. PDX provides a new option for salvage therapy or a bridge to AHSCT in patients with refractory or relapsed ENKL. Here we report what we believe to be the first successful use of PDX as a bridge to AHSCT for the treatment of a patient with advanced-stage disseminated ENKL, nasal type, which was refractory to first-line SMILE chemotherapy.

## Case presentation

A 29-year-old Asian man experienced rhinorrhea, intermittent fever up to 40 ºC (104 °F) two to three times per week, and multiple progressive non-itchy papules on his face and trunk since September 2015. He was treated for chronic atopic dermatitis and chronic sinusitis without obvious improvement after multiple courses of antibiotics since April 2016.

**Fig. 1:**
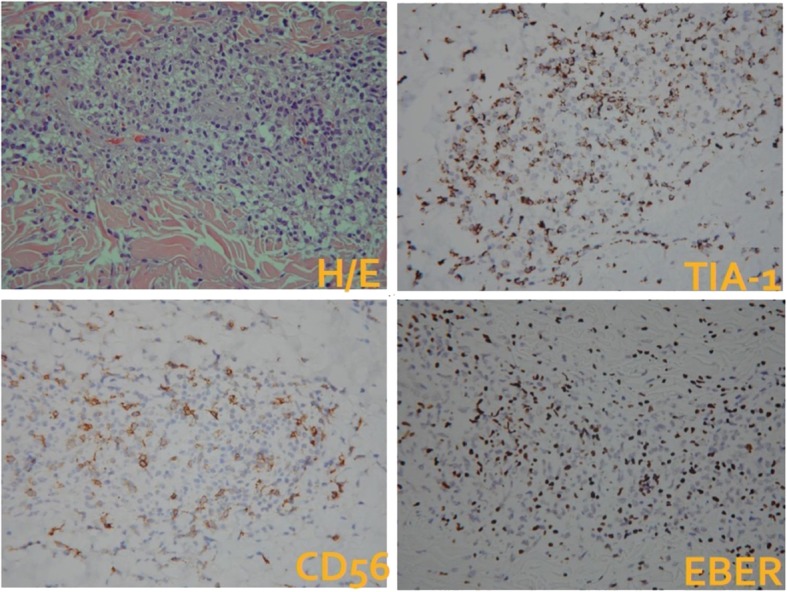
The scattered Epstein–Barr virus-encoded small RNA-positive small lymphocytes were also positive for CD3, CD56, and TIA-1 stains and negative for CD20. *EBER* Epstein–Barr virus-encoded small RNA, *H/E* hematoxylin and eosin

In February 2017, a nasopharyngoscopy revealed necrotic tissue in bilateral nasal cavities and septal perforation. Biopsies were completed for both nasal and skin lesions. The pathology of the skin lesions revealed many small lymphocytic infiltrates in the perivascular area with positive EBV-encoded small RNA (EBER) stain. The scattered EBER-positive small lymphocytes were also positive for CD3, CD56, and TIA-1 stains and negative for CD20. Numerous reactive T cells were also noted to be positive: CD2, CD3, CD4, CD5, CD7, and CD8 (Fig. 1); nasal lesions showed a similar picture. A bone marrow aspirate and biopsy showed 20% small lymphoid cells with special stains similar to skin lesions. A whole-body positron emission tomography (PET)/computed tomography (CT) scan revealed increased fluorodeoxyglucose (FDG) uptake in his bilateral nasal cavity, bilateral neck level I and II lymph nodes, and bilateral axillary regions. EBV polymerase chain reaction (PCR) for peripheral blood showed a viral load of 15,174 copies/mL. He was diagnosed as having ENKL, nasal type, with an Ann Arbor clinical stage of IV.

In March 2017, he underwent systemic chemotherapy with the SMILE regimen (etoposide 100 mg/m^2^ on days 2–4; ifosfamide 1500 mg/m^2^ on days 1–3; mesna 900 mg/m^2^ on days 2–4; methotrexate 2000 mg/m^2^ on day 1; dexamethasone 40 mg on days 1–4; and L-asparaginase 6000 U/m^2^ on days 8, 10, 12, 14, 16, 18, and 20). Cycles were repeated every 28 days. He received two cycles of SMILE, each complicated by intermittent fever after neutropenic recovery, progressive skin lesions, and rebounding EBV viremia during the chemotherapy window. At this point, his ENKL was noted to be primary refractory to the SMILE regimen; therefore, he received two cycles of PDX 30 mg/m^2^ on days 1, 8, 15, 22, 29, and 36 in a PDX clinical trial. After two cycles of PDX, a partial remission of skin lesions and complete remission of nasal lesions, neck lymphadenopathies, and bone marrow were documented.

**Fig. 2:**
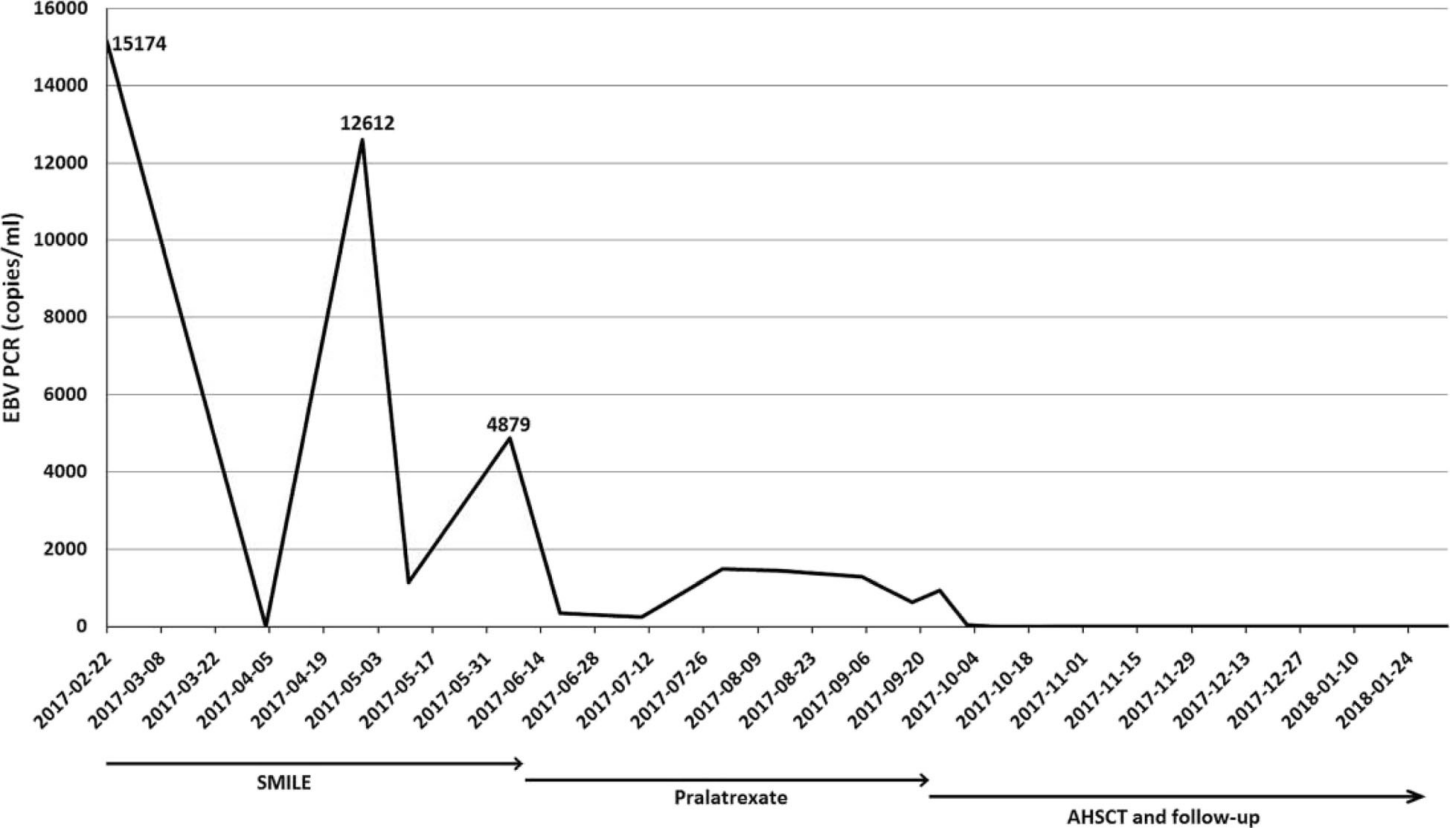
Epstein–Barr virus viremia showed significant decrease at follow-up. *AHSCT* allogeneic hematopoietic stem cell transplantation, *EBV* Epstein–Barr virus, *PCR* polymerase chain reaction, *SMILE* dexamethasone, methotrexate, ifosfamide, L-asparaginase, and etoposide

**Fig. 3:**
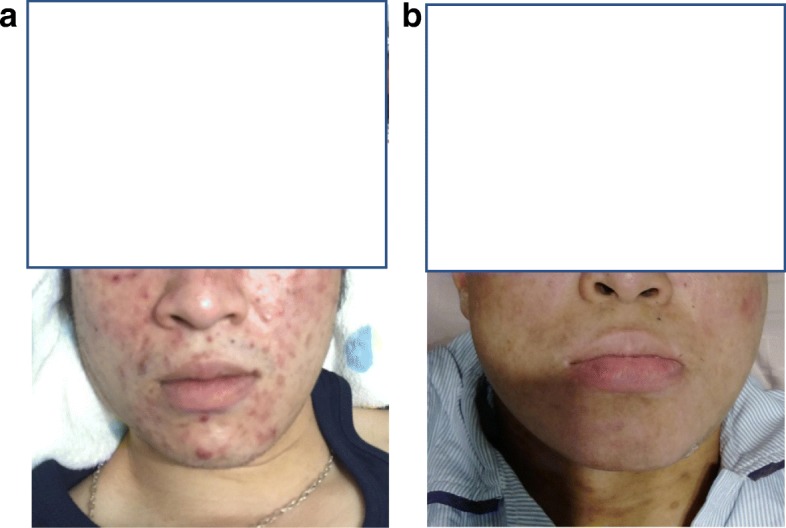
Skin lesions of face and trunk at diagnosis (**a**) and after allogeneic hematopoietic stem cell transplantation (**b**) revealed complete remission

**Fig. 4:**
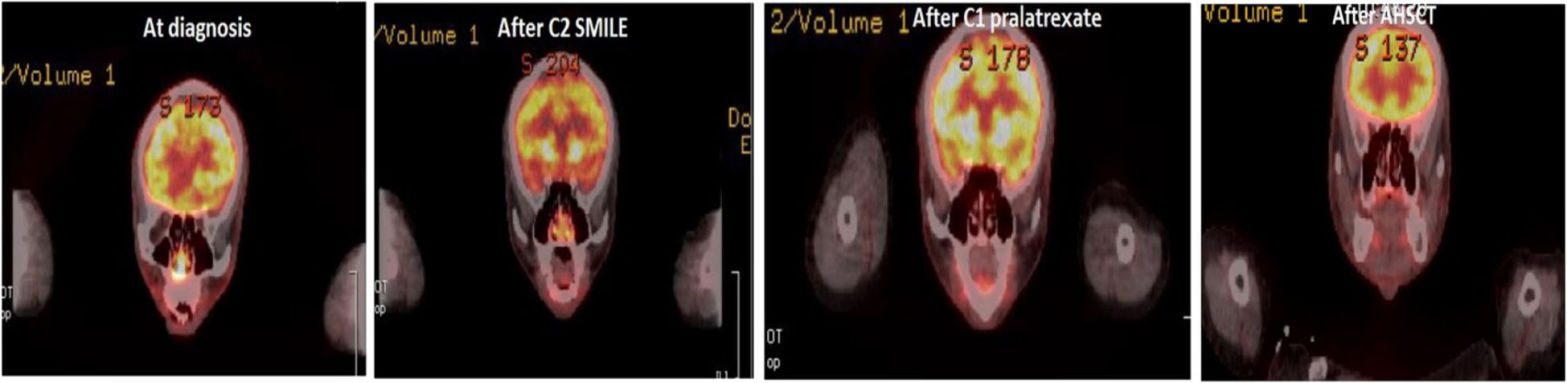
Positron emission tomography-computed tomography scans for nasal lesions and lymphadenopathies showed complete metabolic response after one cycle of pralatrexate and maintained the response after allogeneic hematopoietic stem cell transplantation. *AHSCT* allogeneic hematopoietic stem cell transplantation, *SMILE* dexamethasone, methotrexate, ifosfamide, L-asparaginase, and etoposide

He then underwent a matched unrelated AHSCT. After AHSCT, his disease achieved complete remission. Follow-up of EBV viremia showed significant decrease (Fig. 2). The skin lesions on our patient’s face and trunk also revealed complete remission (Fig. 3). A follow-up PET-CT scan of his nasal lesions and lymphadenopathies showed complete metabolic response after one cycle of PDX and maintained complete response (CR) after AHSCT (Fig. 4). He suffered from progressive acute graft-versus-host disease (aGVHD) with overall grade III and refractory to steroid (2 mg/kg per day) since January 2018. After we administered tocilizumab and sirolimus to our patient, aGVHD was gradually controlled. However, local recurrence of left nasal cavity without new lymphadenopathy, skin involvement, and bone marrow invasion was noticed in March 2018. Local radiotherapy with 5000 cGy/25 fractions to the relapse lesion and 4500 cGy/25 fractions to bilateral nasal cavities were administered. PET-CT revealed complete remission of nasal cavity in September 2018. However, one ulcerative skin lesion on his right forearm was noted. A biopsy showed ENKL, nasal type. Therefore, concurrent chemoradiotherapy (CCRT) with gemcitabine, Leunase (L-asparaginase), and oxaliplatin (GeLOX) regimen for four cycles and local radiotherapy with 5000 cGy/20 fractions to his right forearm lesion were administered until December 2018. The skin lesion revealed good healing without progression. We also gave low-dose nivolumab (20 mg) every month for maintenance therapy until January 2020. A follow-up of PET-CT, EBV viremia, skin lesion, and bone marrow showed no evidence of disease relapse.

## Discussion and conclusions

The treatment for advanced-stage disseminated ENKL, nasal type, is very challenging for clinicians. Because ENKL cells are associated with a high expression of P-glycoprotein, which causes multidrug resistance and could contribute to the poor response associated with anthracycline-based chemotherapy regimens, we opted to use the SMILE regimen in our patient [8]. Once ENKL is deemed primary refractory to first-line chemotherapy, the prognosis is very poor [5, 6]. Although AHSCT is a potentially curative therapy with the possibility of disease relapse prevention, salvage chemotherapy with an effective treatment as a bridge to AHSCT is not well understood, especially for advanced-stage nasal type ENKL. Most patients often die of disease progression, infections, or associated complications after failure of first-line chemotherapy.

Our patient received a total of two cycles of SMILE chemotherapy and was unfortunately considered primary refractory to this therapy. He demonstrated a potentially excellent therapeutic effect of PDX in a patient with stage IV ENKL, nasal type, refractory to first-line SMILE. Through a bridge of PDX, he ultimately underwent AHSCT, leading to complete remission of disease. This is the first presentation to explore PDX as a successful bridge to AHSCT in a patient with advanced-stage ENKL refractory to first-line SMILE chemotherapy.

Circulating EBV DNA levels have been observed to be important for prognostication in NKTCL [3]. The National Comprehensive Cancer Network (NCCN) now recommends monitoring EBV DNA load and calculation of Prognostic Index of Natural Killer Lymphoma (PINK) or Prognostic Index of Natural Killer Lymphoma with Epstein–Barr Virus DNA (PINK-E) as part of the initial workup for ENKL. These recommendations are based on a retrospective study that analyzed EBV DNA in 328 patients and determined that a detectable EBV DNA titer was an independent prognostic factor for overall survival (OS) [16].A prospective observational study in patients with NKTCL treated with an asparaginase-based therapy determined that pretreatment EBV DNA levels correlated with a lower rate of CR, and post-treatment EBV DNA levels were a prognostic factor for both progression-free survival (PFS) and OS (in patients with CR, post-treatment EBV DNA positivity correlated with inferior PFS and OS; *p* < 0.0001) [17]. Similarly, another study found that higher levels of pretreatment EBV DNA predicted poor response rates to the SMILE regimen, indicating that the pretreatment EBV DNA level may be a positive predictive marker for response after asparaginase-based treatment [18]. If these findings are validated in future prospective clinical trials, patients with high pretreatment EBV DNA levels may benefit from more intensive treatments or novel drugs other than asparaginase-based therapy. In our patient, the EBV viral load rebounded during failure to SMILE with progressive generalized symptoms and skin lesions, but showed a significant downregulation of plasma EBV viral titer from the point of PDX therapy. An undetectable plasma EBV viral titer was noticed after AHSCT. The improvement of EBV viremia in our patient was also compatible with continuous clinical disease control.

In summary, our case demonstrates the successful use of PDX as a successful bridge to AHSCT, which played an integral role in our patient’s overall outcome, leading to complete disease remission. Due to the rarity of ENKL, it is unlikely that randomized trials will be conducted; however, based on our case’s outcome, we believe that this therapeutic schema warrants further evaluation to expand treatment options available to patients with advanced relapsed/refractory nasal type ENKL.

## Data Availability

The datasets used and/or analyzed during the current study are available from the corresponding author on reasonable request.

## Abbreviations

aGVHD: Acute graft-versus-host disease
AHSCT: Allogeneic hematopoietic stem cell transplantation
CCRT: Concurrent chemoradiotherapy
CR: Complete response
CT: Computed tomography
EBER: Epstein–Barr virus-encoded small RNA
EBV: Epstein–Barr virus
ENKL: Extranodal natural killer/T cell lymphoma
FDG: Fluorodeoxyglucose
GeLOX: Gemcitabine, Leunase (L-asparaginase), and oxaliplatin
HSCT: Hematopoietic stem cell transplantation
NCCN: National Comprehensive Cancer Network
NK: Natural killer
NKTCL: Natural killer/T cell lymphoma
OS: Overall survival
PCR: Polymerase chain reaction
PDX: Pralatrexate
PET: Positron emission tomography
PFS: Progression-free survival
PINK: Prognostic Index of Natural Killer Lymphoma
PINK-E: Prognostic Index of Natural Killer Lymphoma with Epstein–Barr Virus DNA
PTCL: Peripheral T cell lymphoma
SMILE: Dexamethasone, methotrexate, ifosfamide, L-asparaginase, and etoposide
